# Effect of the *In Situ* Screw Implantation Region and Angle on the Stability of Lateral Lumbar Interbody Fusion: A Finite Element Study

**DOI:** 10.1111/os.13312

**Published:** 2022-06-03

**Authors:** Guangye Zhu, Zhihua Wu, Zhichao Fang, Peng Zhang, Jiahui He, Xiang Yu, Zhilin Ge, Kai Tang, De Liang, Xiaobing Jiang, Ziyang Liang, Jianchao Cui

**Affiliations:** ^1^ 1st Clinical Medical College Guangzhou University of Chinese Medicine Guangzhou China; ^2^ Department of Spinal Surgery 1st Affiliated Hospital of Guangzhou University of Chinese Medicine Guangzhou China; ^3^ Department of Orthopedics The Second Xiangya Hospital of Central South University Changsha China

**Keywords:** Finite element analysis, *In situ* screw, Lateral lumbar interbody fusion, Stability, Surgical procedures

## Abstract

**Objective:**

To investigate the effect of the *in situ* screw implantation region and angle on the stability of lateral lumbar interbody fusion (LLIF) from a biomechanical perspective.

**Methods:**

A validated L2‐4 finite element (FE) model was modified for simulation. The L3‐4 fused segment undergoing LLIF surgery was modeled. The area between the superior and inferior edges and the anterior and posterior edges of the vertebral body (VB) is divided into four zones by three parallel lines in coronal and horizontal planes. *In situ* screw implantation methods with different angles based on the three parallel lines in coronal plane were applied in Models A, B, and C (A: parallel to inferior line; B: from inferior line to midline; C: from inferior line to superior line). In addition, four implantation methods with different regions based on the three parallel lines in horizontal plane were simulated as types 1–2, 1–3, 2–2, and 2–3 (1–2: from anterior line to midline; 1–3: from anterior line to posterior line; 2–2: parallel to midline; 2–3: from midline to posterior line). L3‐4 ROM, interbody cage stress, screw‐bone interface stress, and L4 superior endplate stress were tracked and calculated for comparisons among these models.

**Results:**

The L3‐4 ROM of Models A, B, and C decreased with the extent ranging from 47.9% (flexion‐extension) to 62.4% (lateral bending) with no significant differences under any loading condition. Types 2–2 and 2–3 had 45% restriction, while types 1–2 and 1–3 had 51% restriction in ROM under flexion‐extension conditions. Under lateral bending, types 2–2 and 2–3 had 70.6% restriction, while types 1–2 and 1–3 had 61.2% restriction in ROM. Under axial rotation, types 2–2 and 2–3 had 65.2% restriction, while types 1–2 and 1–3 had 59.3% restriction in ROM. The stress of the cage in types 2–2 and 2–3 was approximately 20% lower than that in types 1–2 and 1–3 under all loading conditions in all models. The peak stresses at the screw‐bone interface in types 2–2 and 2–3 were much lower (approximately 35%) than those in types 1–2 and 1–3 under lateral bending, while no significant differences were observed under flexion‐extension and axial rotation. The peak stress on the L4 superior endplate was approximately 30 MPa and was not significantly different in all models under any loading condition.

**Conclusions:**

Different regions of entry‐exit screws induced multiple screw trajectories and influenced the stability and mechanical responses. However, different implantation angles did not. Considering the difficulty of implantation, the ipsilateral‐contralateral trajectory in the lateral middle region of the VB can be optimal for *in situ* screw implantation in LLIF surgery.

## Graphical abstract

Different angles of entry‐exit screw influenced the stability and mechanical responses of the fused segment. Ipsilateral‐contralateral trajectory in lateral middle region of the VB can be an optimized selection for *in situ* screw implantation in LLIF.

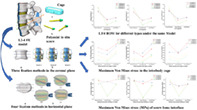

## Introduction

Over the past 15 years, significant interest has arisen in minimally invasive anterolateral approaches to the lumbar spine^1^. Lateral lumbar interbody fusion (LLIF) is one of the most widely used procedures to treat lumbar degenerative disease (LDD). Oblique lumbar interbody fusion (OLIF) was first reported in 2012^2^ and is an anterior psoas surgical approach embodying a paradigm shift in LLIF. This surgical procedure does not require a posterior approach, including no need for laminectomy, facetectomy, or stripping of the spinal musculature^3^. Therefore, as a minimally invasive surgery (MIS), LLIF has attracted the attention of spinal surgeons due to its fewer postoperative neurological complications and a lower risk of pseudoarthrosis and axial pain.

Successful fusion is conducive to a satisfactory clinical outcome. Since the anterior and posterior longitudinal ligaments are preserved, stand‐alone LLIF (without supplemental internal fixation) was once considered to have the ability to stabilize the mobile segment^4^. However, a numerical approach by Calvo‐Echenique *et al*. reported that spinal movement with stand‐alone LLIF could compromise intervertebral fusion and might present a higher risk of cage subsidence^5^. Clinically, Marchi *et al*. reported a 1‐year follow‐up after stand‐alone LLIF; 70% of the cases had mild to moderate cage subsidence, and 30% were considered severe subsidence^6^. Hence, to achieve sufficient stability of the fused segment, supplemental internal fixation is often required.

Supplemental internal fixation can be performed in different ways. Spinal surgeons accustomed to traditional posterior lumbar interbody fusion (PLIF)/transforaminal lumbar interbody fusion (TLIF) surgery might prefer posterior pedicle screw fixation. However, previous studies have demonstrated that lateral instrumentation could provide similar stability of the fused segment and reasonable biomechanical protection of the interbody cage and endplate compared to pedicle screws^7^, ^8^. Fogel *et al*. showed that lateral stabilization added to the vertebra and the spinous process could achieve stiffness under all loading conditions, similar to pedicle screws^7^. Zhang *et al*. reported that the lateral plate increased stiffness under bending and axial rotation and reduced cage stress in all motion modes^9^. Therefore, some scholars have tried to use anterolateral *in situ* screws as supplemental instrumentation in LLIF^10^, ^11^. Furthermore, this procedure was revealed to be more minimally invasive than posterior fixation in clinical practice^12^, ^13^. The placement of anterolateral *in situ* screws avoids damage to the posterior bony elements, conducive to withstanding greater compressive loads, resulting in an important bearing function due to its mechanical properties^14^. Moreover, it could be performed in the original lateral position without shifting the patient into the prone position, which improves the operation efficiency and reduces the operation trauma.

To investigate the anatomically safe zones relative to the disc spaces for the prevention of nerve injuries during the lateral retroperitoneal transpsoas approach, Uribe *et al*. put forward a new division theory in 2010^15^. In their study, the VB was laterally divided from anterior to posterior into four zones (Zones I‐IV). They found that the safe anatomical zones at the disc spaces from L1‐2 to L3‐4 were at the midpoint of Zone III and that the safe anatomical zone at the L4‐5 disc space was at the Zone II to Zone III demarcation. *In situ* screw implantation and disc space preparation are both performed through the lateral retroperitoneal approach in LLIF surgery, and both have a risk of nerve injuries. Therefore, referring to the classification proposed by Uribe *et al*., the area between the anterior and posterior edges and the superior and inferior edges of the vertebral body (VB) is divided into four zones by three parallel lines in the coronal and horizontal planes. Theoretically, anterolateral *in situ* screws could be implanted in different regions and at different angles. However, to date, no study has been reported on whether different *in situ* screw implantation regions and angles have any effect on segmental stability. Therefore, the goal of this study was (i) to introduce screw‐setting strategies for anterolateral *in situ* screws following LLIF and (ii) to explore the biomechanical effect on different screw‐setting strategies following LLIF using finite‐element analysis (FEA). It was hypothesized that different implantation regions and angles of screws possess various degrees of mechanical stability and protective effects.

## Materials and Methods

### Model Construction

**Fig. 1:**
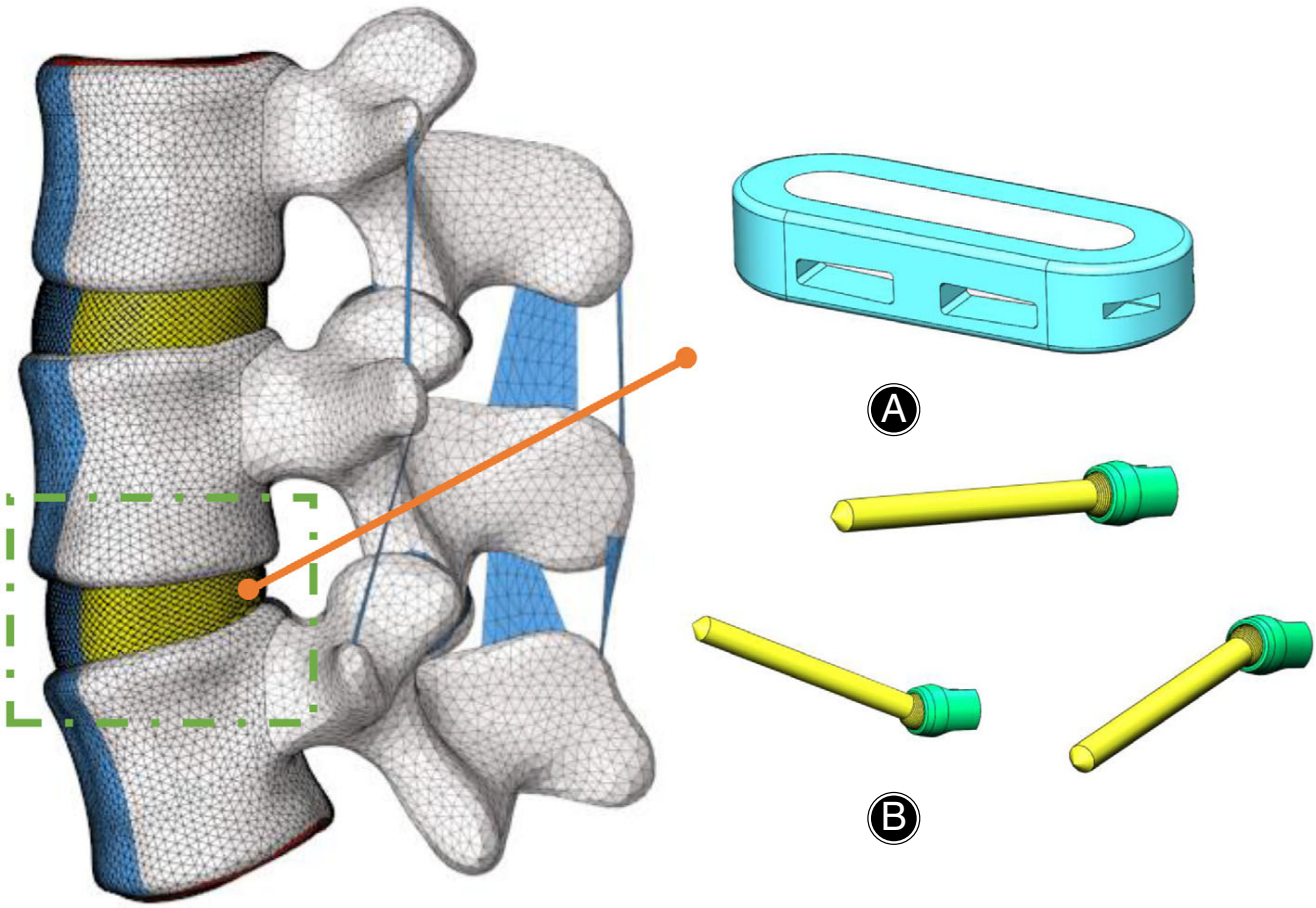
Finite element model of the L2–L4 spine segment. The designed lateral cage (A) was placed at the L3‐4 segment. The designed polyaxial *in situ* screw (B) was placed at the L3 and L4 vertebrae

A 3D FE model of L2‐L4 was constructed in this study (Figure 1). The imaging data were obtained from computed tomography (CT) scans (slice thickness, 1 mm) of a male volunteer. The 3D geometric structure was constructed using Mimics (version 19.0; Materialize, Inc., Leuven, Belgium), which transformed the DICOM images into a digital model. The model was smoothed, amended, and spherized with Geomagic Studio (version 2015; Geomagic, SC, USA). The cancellous bone, zygapophyseal cartilage, and intervertebral disc were used to generate a solid model in SolidWorks CAD software (version 2017; SolidWorks Corp, Dassault Systèmes, Concord, MA). The gap in the zygapophyseal joint was approximately simulated using CT images. The intervertebral disc was partitioned into the annulus fibrosus and nucleus pulposus; the nucleus pulposus was defined as 43% of the total disc volume and was located slightly posterior to the center of the disc^16^, ^17^, ^18^, ^19^.

Then, preprocessing FE software Simlab (version 2019.2, Altair Engineering, USA) was used to construct the spinal ligaments. The anterior/posterior longitudinal ligament (ALL/PLL), ligamentum flavum (LF), interspinous ligament (ISL), supraspinous ligament (SSL), and intertransverse ligament (ITL) were constructed in the FE model.

The vertebra consisted of the cortical layer with a thickness of 1 mm^20^ and the inner cancellous bone. At both ends of each vertebra, cartilaginous endplates were simulated with a thickness of 0.8 mm^21^. These spinal ligaments were defined as four‐node shell elements with different thicknesses^22^. Additionally, Simlab software was used to set the material properties of the lumbar spine components. The material properties have previously been described in the literature, as specified in Supplementary material. Two‐node 3D truss element (T3D2s) properties were assigned to fibers of the annulus fibrosus. Four reticular fiber layers were added to the ground substance at an angle between 24° and 45°^23^. The contact between adjacent facet joint surfaces was defined as the coefficient of friction and was set at 0.1^24^. Each lumbar spine component was created with 2D mesh and 3D volume mesh in Simlab preprocessing software.

### Boundary and Loading Conditions

The inferior surface of the L4 vertebra was completely constrained in all directions, and the loading condition was applied to the superior surface of the L2 vertebra. Utilizing an approach similar to that of Shim *et al*.^25^, a 500 N axial compressive preload was set, and a pure moment of 7.5 Nm was applied to simulate the model in six directions: (1) flexion (Flx); (2) extension (Ext); (3) left bending (LB); (4) right bending (RB); (5) left rotation (LR); and (6) right rotation (RR). The applied load in this study was deemed sufficient to generate maximum physiological motion but insufficient to harm the specimens. ABAQUS 2016 software (version 2016, SIMULIA, Inc., USA) was used for these analyses.

### FE Model Validation

The kinematic behavior of the FE model was verified under the conditions of flexion, extension, lateral bending, and axial rotation. The range of motion (ROM) and intradiscal pressure (IDP) were the parameters chosen for validation. To validate the present model, a few criteria were applied. They were the mean absolute error (MAE) and the root mean squared error (RMSE)^26^, ^27^, ^28^ .RMSE and MAE are methods that attempt to determine the relationships between input variables and one or more response variables. These criteria are as follows:

(1) $$\mathrm{MAE}=\frac{\sum_{i=1}^{n}|y_{i}-x_{i}|}{n}$$

(2) $$\text{RMSE}=\sqrt{\frac{1}{n}\sum_{k=1}^{n}{\left|y_{i}-x_{i}\right|}^{2}}$$

where $y_{i}$ are predicted values that were obtained from the finite element model, $x_{i}$ are actual values, and n are the numerical data that were analyzed, as shown in Formulas 1 and 2. To compare the various errors in predicting outputs in this case, a lower score for MAE and RMSE means better matching of the finite element model and experimental sample.

### Stress Sensitivity Analysis

For the sensitivity analysis of the material properties, the intact FE models in this study were tested. High‐value and low‐value models were created from the linearized basic model by a 25% linear increase and decrease, respectively. ROM and IDP were chosen for testing in this study. Since the stress and strain results were focused on the L3‐L4 level, the ROM and LDP values at the L3‐L4 level obtained by the linearized basic, high‐value, and low‐value models were compared. The objective of the stress sensitivity analysis was to provide insight into the overall effect of material property variations on biomechanical behavior.

### FE Model with Implants

The intact lumbar spine model was modified to simulate instrumented LLIF with different types of internal fixation. In each group, intervertebral cage fusion was modeled at the level of the L3‐4 segment (Figure 1A). The cage inserted laterally was box‐shaped (45 mm in length, 18 mm in width, 9 mm in height), with an 8° incline between the superior and inferior surfaces (DePuy Synthes Spine, Inc., Raynham, MA). The cage was centered on the middle sagittal plane in the disc space. In addition, we established a polyaxial screw, which consists of a pedicle screw with a spherical cap through a universal sleeve. One end of the universal sleeve was set as a “U” shape, and the other end was set as a spherical socket with a hole, so the screw could be fixed in multiple directions (Figure 1B), resulting in multiple screw trajectories.

**Fig. 2:**
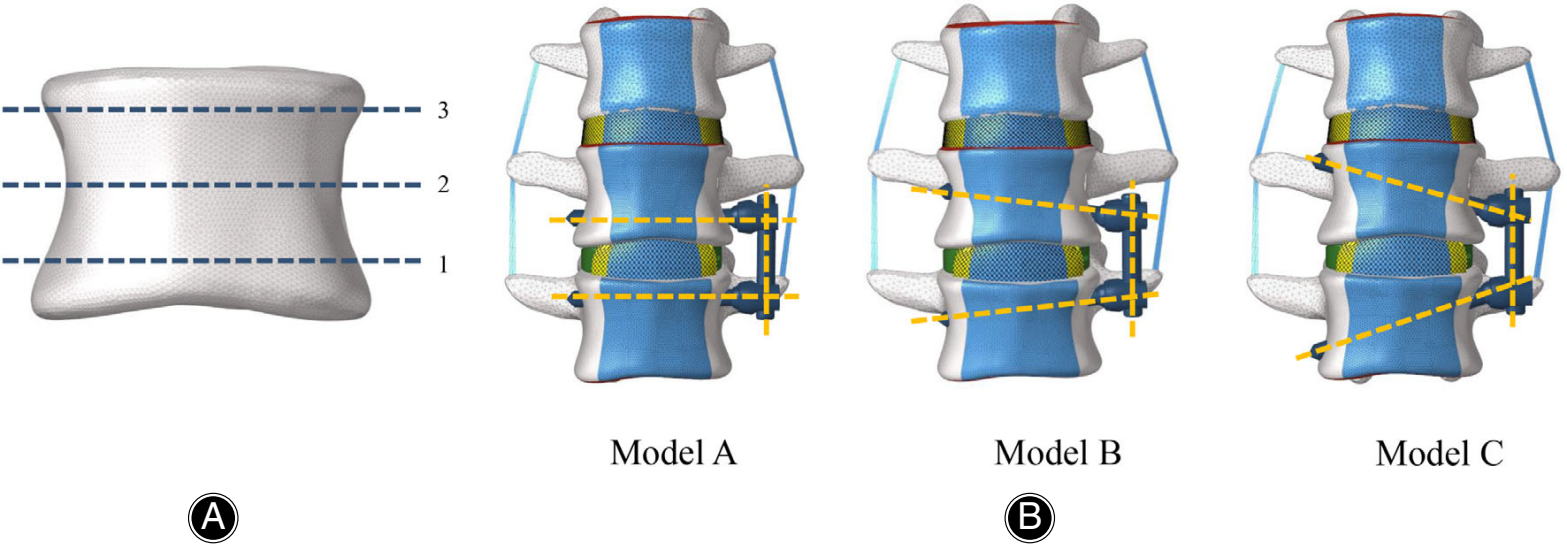
In the coronal plane, Line 1 was adjacent to the inferior endplate, Line 2 was in the middle of the VB, and Line 3 was adjacent to the superior endplate (A). Three fixation methods based on different angles were defined: the angle of the screws in Model A fixation was parallel to Line 1, the distal end of the screws reached Line 2 in Model B fixation, and the distal end of the screws reached Line 3 in Model C fixation

**Fig. 3:**
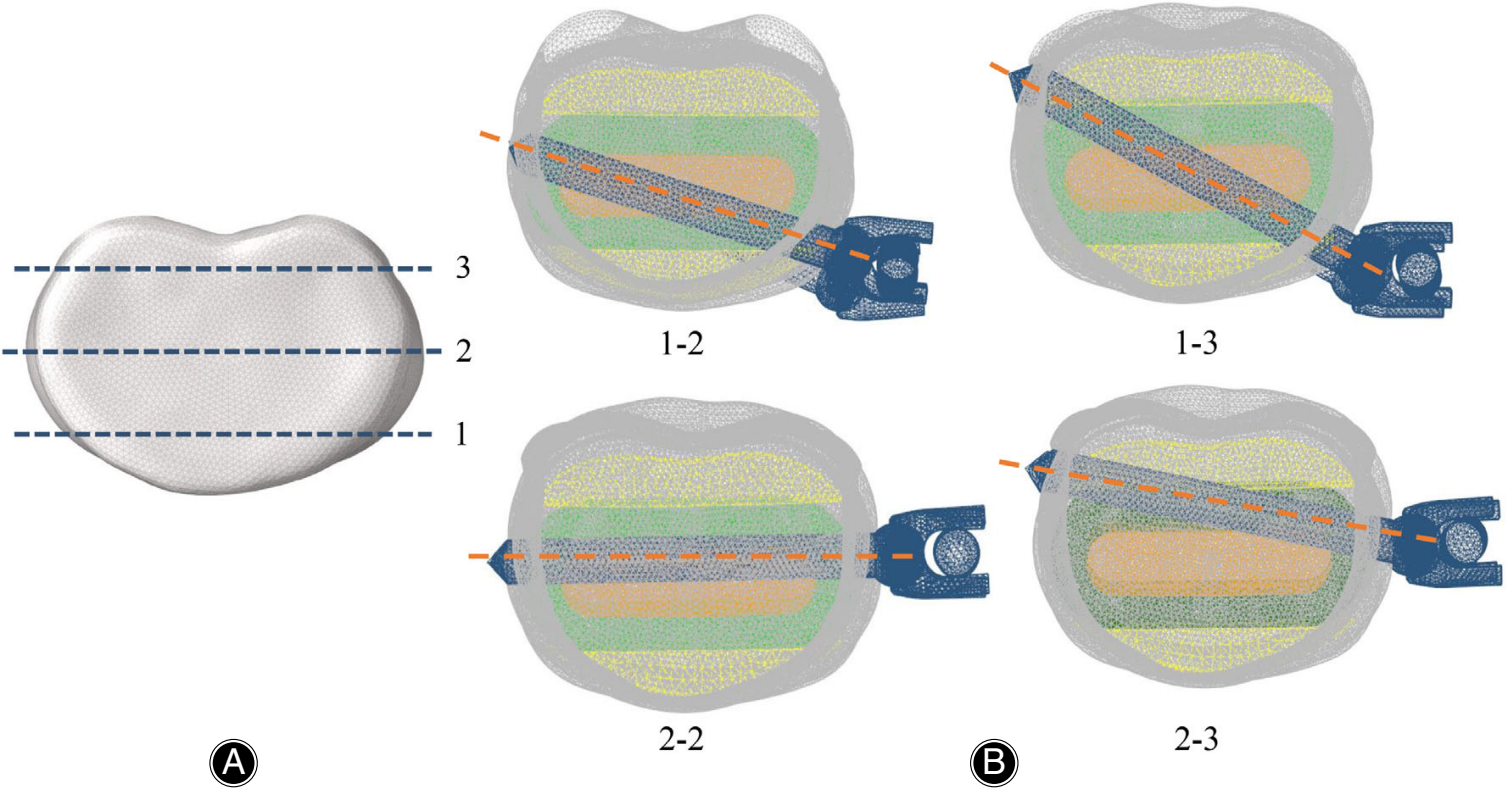
(A) In the horizontal plane, Line 1 was adjacent to the anterior edge of the VB, Line 2 was in the middle of the VB, and Line 3 was adjacent to the posterior edge of the VB. (B) Four fixation methods based on different implantation regions were simulated. Type 1–2: screws were implanted from Line 1 of one side of VB to Line 2 of the other side of VB. Type 1–3: screws were implanted from Line 1 of one side of VB to Line 3 of the other side of VB. Type 2–2: screws were implanted from Line 2 of one side to the other side of VB. Type 2–3: screws were implanted from Line 2 of one side of VB to Line 3 of the other side of VB

To explore the screw‐setting strategies for the anterolateral *in situ* screws, three parallel lines were designed in the coronal and horizontal planes, and each plane of the VB was consequently divided into four zones. In the coronal plane, Line 1 was adjacent to the inferior endplate, Line 2 was in the middle of the VB, and Line 3 was adjacent to the superior endplate (Figure 2A). Similarly, in the horizontal plane, Line 1 was adjacent to the anterior edge of the VB, Line 2 was in the middle of the VB, and Line 3 was adjacent to the posterior edge of the VB (Figure 3A). The distance between Line 1 and Line 2 was equal to that between Line 2 and Line 3 in the coronal and horizontal planes. In the coronal plane, we defined three different fixation methods: the angle of the *in situ* screws in Model A fixation was parallel to Line 1, the distal end of the *in situ* screws reached Line 2 in Model B fixation, and the distal end of the *in situ* screws reached Line 3 in Model C fixation (Figure 2B). The entry points of Models A, B, and C were the same. In the horizontal plane, we simulated four fixation methods. When an *in situ* screw was inserted from the front (Line 1) of one side of the VB and penetrated the cortex from the middle (Line 2) of the other side of the VB, we defined the fixation method as type 1–2. Similarly, after penetrating the cortex from the posterior (Line 3) of the other side of the VB, we defined the fixation method as type 1–3. When an *in situ* screw was inserted from the middle (Line 2) of one side of the VB and penetrated the cortex from Line 2 and Line 3, we defined it as 2–2 and 2–3, respectively (Figure 3B).

The internal fixation and cage implants were reconstructed in SolidWorks and fitted closely to the surrounding structures. In these models, the diameter of the pedicle screws was 6.0 mm, and the length of the screws was set to reach the anterior or lateral cortex of the VB. All screws were fixed to the vertebral bodies without allowing relative motion, which was achieved by assigning the contact surfaces to be tied in Simlab software. The rods connecting the screws were selected for lofting and reconstruction to ensure an exact fit. Pedicle screws and rods were defined using a “tie” constraint at the interfaces. A finite sliding algorithm with a coefficient of friction of 0.2 was defined between the cage and L4 superior interface to allow for any small relative displacements between the two contacting surfaces. The screwrod and cage were also tested for mesh convergence, and internal fixation was simulated as a homogeneous linear‐elastic material with the material properties of titanium alloy (E = 110 GPa, Poisson's ratio = 0.3) and polyetheretherketone (E = 3.6 GPa, Poisson'sratio = 0.3)^36^.

### Analysis

L3/4 ROM, interbody cage stress (von Mises stress), screw‐bone interface stress, and L4 superior endplate stress were tracked and calculated to compare the models.

## Results

### Model Validation

**Fig. 4:**
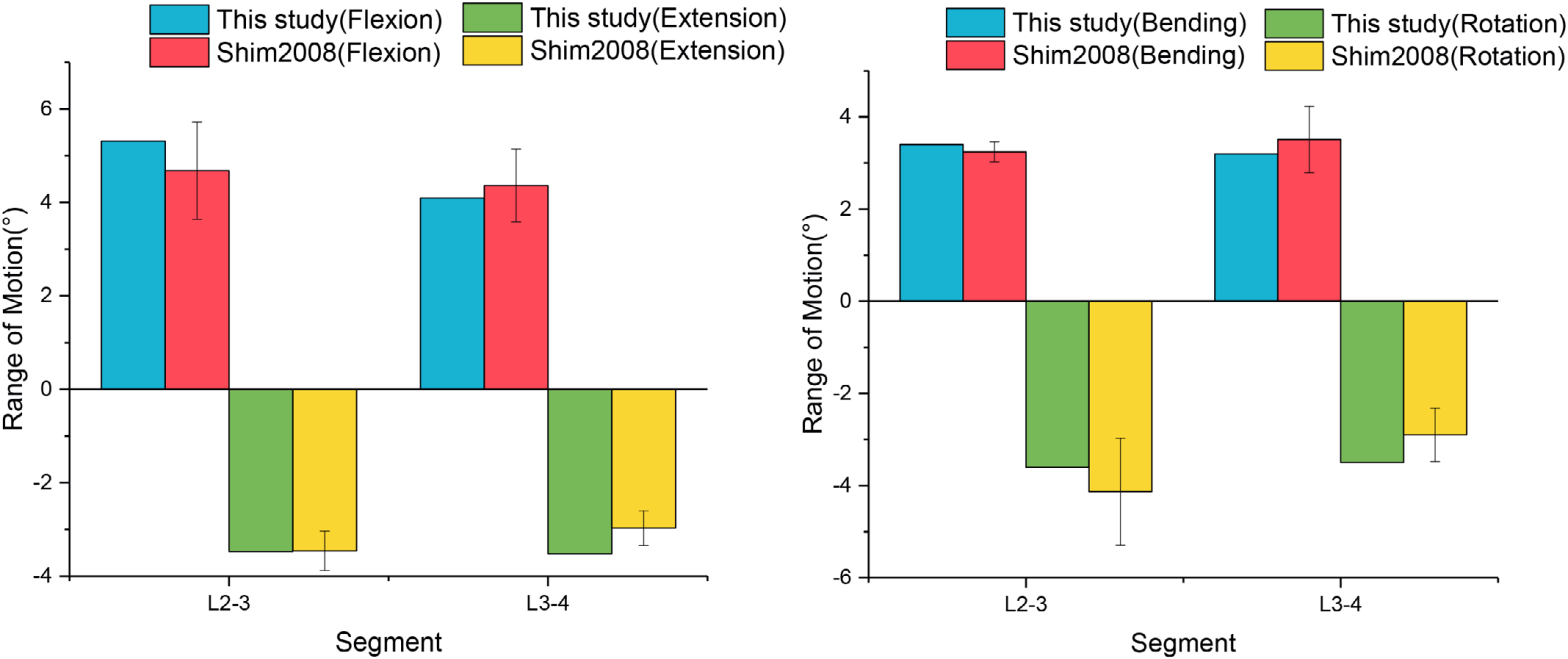
Comparison of L2‐3 and L3‐4 ROM of the intact lumbar spine with previous experimental results

**Fig. 5:**
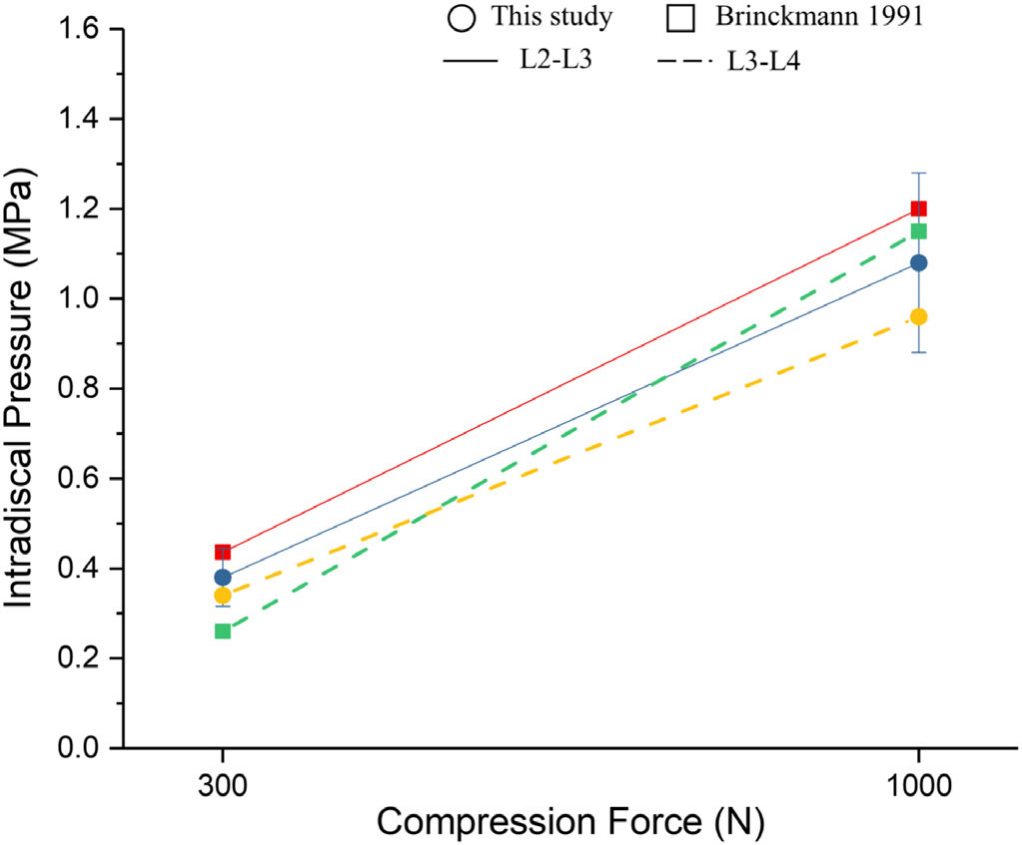
Comparison of L2‐3 and L3‐4 IDP of the intact lumbar spine with previous experimental results

A mesh convergence test indicated that the FE solution used a model with 99,948 nodes and 241,923 elements. A mesh quality assessment in Simlab software showed that 1% of the elements had an aspect ratio of less than 5.0 (maximum 10.0), while 97% and 96% of all shell and solid elements had Jacobian values larger than 0.6 (minimum 0.24). We compared the intact lumbar spine model with a previous cadaverbiomechanical study under the same loading conditions. The ROM values of the L2/3 and L3/4 segments are well‐correlated with the results of Shim *et al*., as shown in Figure 4, ^21^. Under flexion, bending, and rotation, the maximum ROM occurred at L2/3, while the maximum ROM for extension was observed at L3/4. In this study, relative to the experimental and FE values, the MAE (0.50) and RMSE (0.48) values are minor, which indicates that the present FE model possesses a good predictive capacity. Moreover, under 300 N and 1000 N compressive forces of L2/3 IDP, the results are consistent with the results of Brinckmann *et al*.^22^, as shown in Figure 5. All of the above results confirmed the rationality of the model and that it could be further analyzed.

### Stress Sensitivity Analysis

**Fig. 6:**
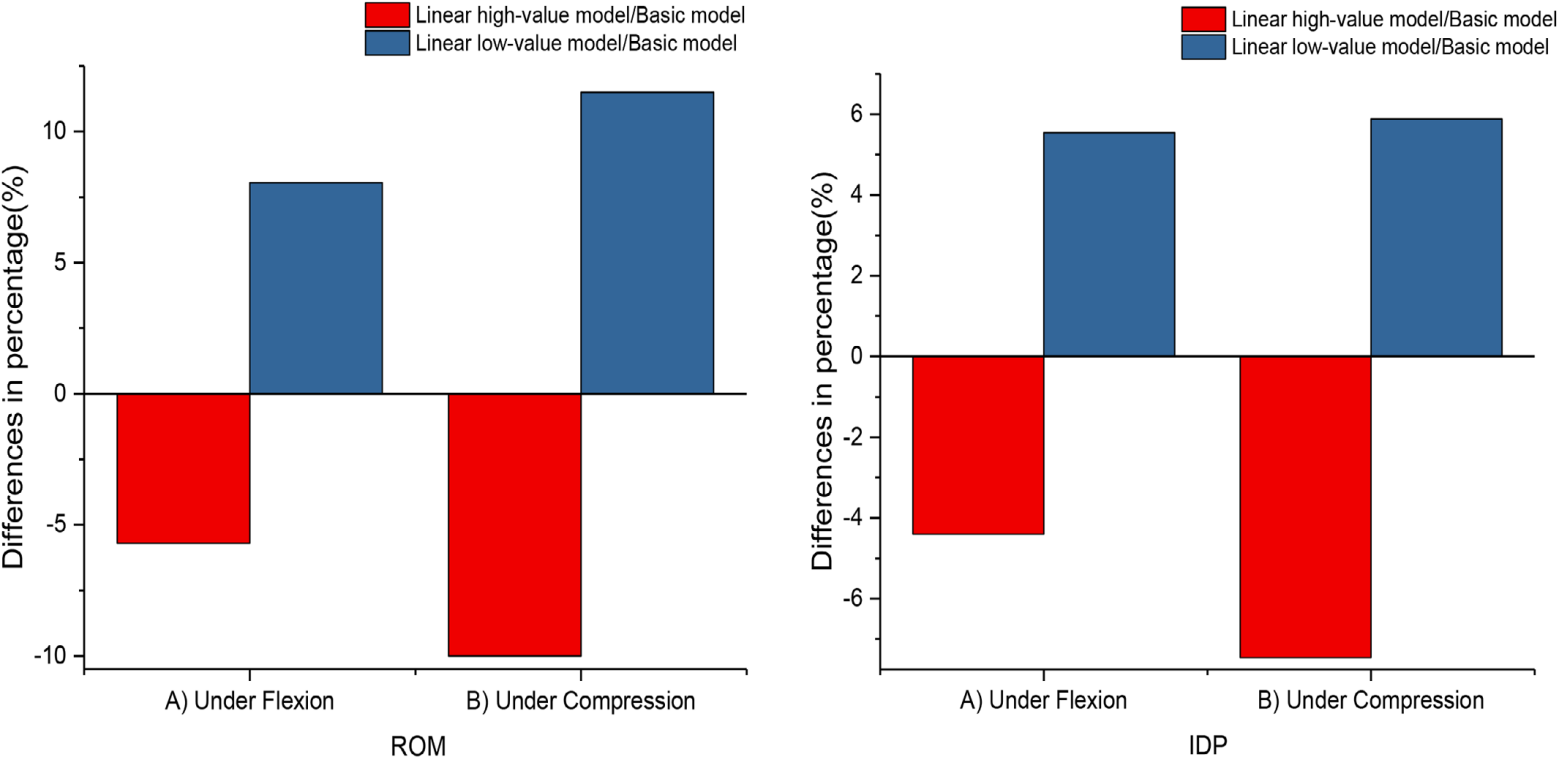
The percentage difference in the ROM and IDP between the linear basic model and the linear low‐value model, and between the linear basic model and the linear high‐value model under flexion and compression

The percentage differences in the ROM and IDP between the linear basic model and the linear low‐value model and between the linear basic model and the linear high‐value model under flexion and compression are shown in Figure 6. In terms of flexion, between the linear basic model and the linear low‐value model, the percent difference in the ROM was 8.1%, and in the IDP, it was 5.5%. Comparing the linear basic model and the linear high‐value model, the percent difference in the ROM was 5.7% and in the IDP it was 4.4%. In terms of compression, between the linear basic model and the linear low‐value model, the percent difference in the ROM was 11.5% and in the IDP it was 5.9%. Comparing the linear basic model and the linear high‐value model, the percent difference in the ROM was 10.0% and in the IDP it was 7.5%.

### ROM

**Fig. 7:**
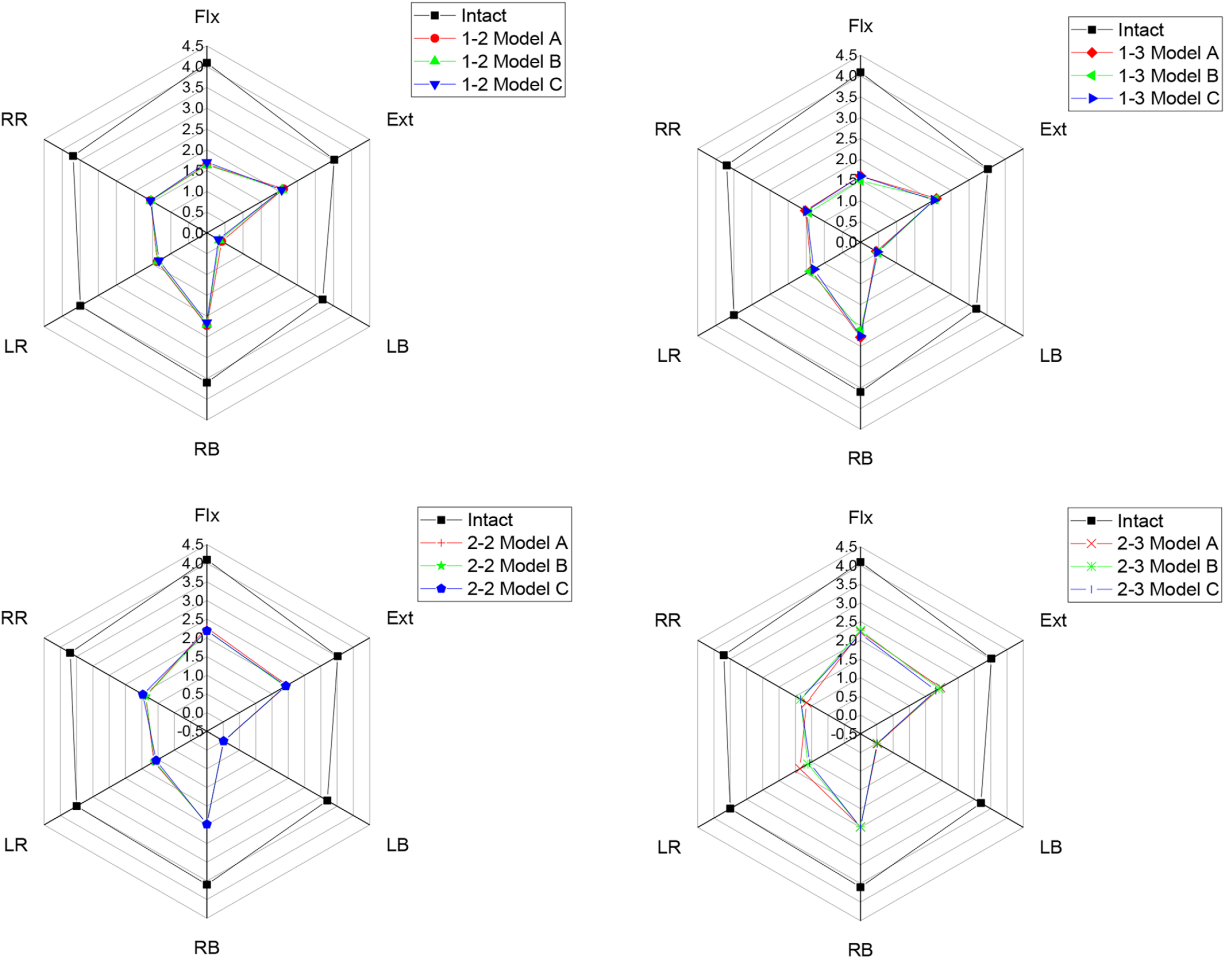
Comparison of L3‐4 ROM for intact and implanted models at the fusion segment

**Fig. 8:**
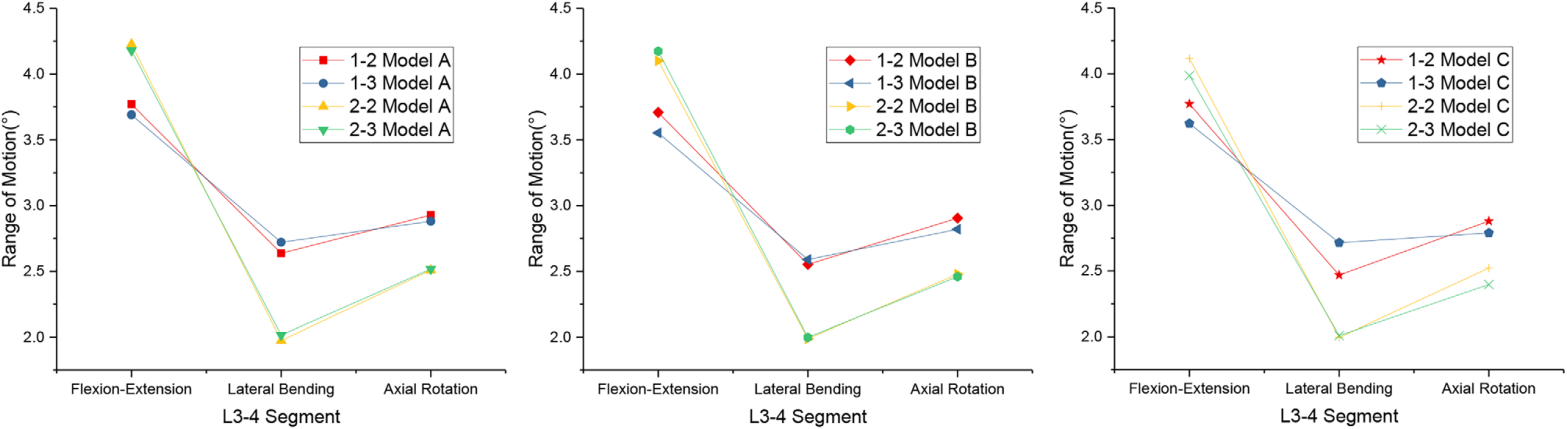
Comparison of L3‐4 ROM for different types under the same model at the fusion segment

Since ROM is a stress sensitivity index, this study compared the ROM among the three models with different angles. Compared with the intact model under all loading conditions, the ROM of Models A, B, and C at L3/4 was significantly reduced, as shown in Figure 7. However, there was no ROM difference in each movement direction among the three models. Therefore, the following section mainly explores the influences of the different implantation regions on the mechanical response of LLIF, as shown in Figure 8.

#### Flexion‐Extension

All models provided the lowest ROM restriction under flexion‐extension. No ROM difference existed among the three models in types 1–2 and 1–3 or types 2–2 and 2–3. However, in Model A, types 1–2 and 1–3 (51% restriction) restricted ROM more than types 2–2 and 2–3 (45% restriction) compared with the intact model, and this trend existed in Models B and C.

#### Lateral Bending

For lateral bending, types 1–2 and 1–3 reduced the ROM of the intact model by 61.2% in Model A, and the ROM was 1.3 times greater than that of types 2–2 and 2–3 (70.6% restriction). There was no significant difference in type 2–2 and type 2–3. This trend also existed in Models B and C. All models had the largest ROM restriction under lateral bending than flexion‐extension and axial rotation.

#### Axial Rotation

Similar to lateral bending, types 2–2 and 2–3 reduced the ROM of the intact model by 65.2% more than types 1–2 and 1–3 (59.3% restriction) in Model A. Similar ROM restrictions existed between types 1–2 and 1–3 and types 2–2 and 2–3 of Model B together with Model C.

### Magnitudes of the Maximum von Mises Stress in the Interbody Cage

**Fig. 9:**
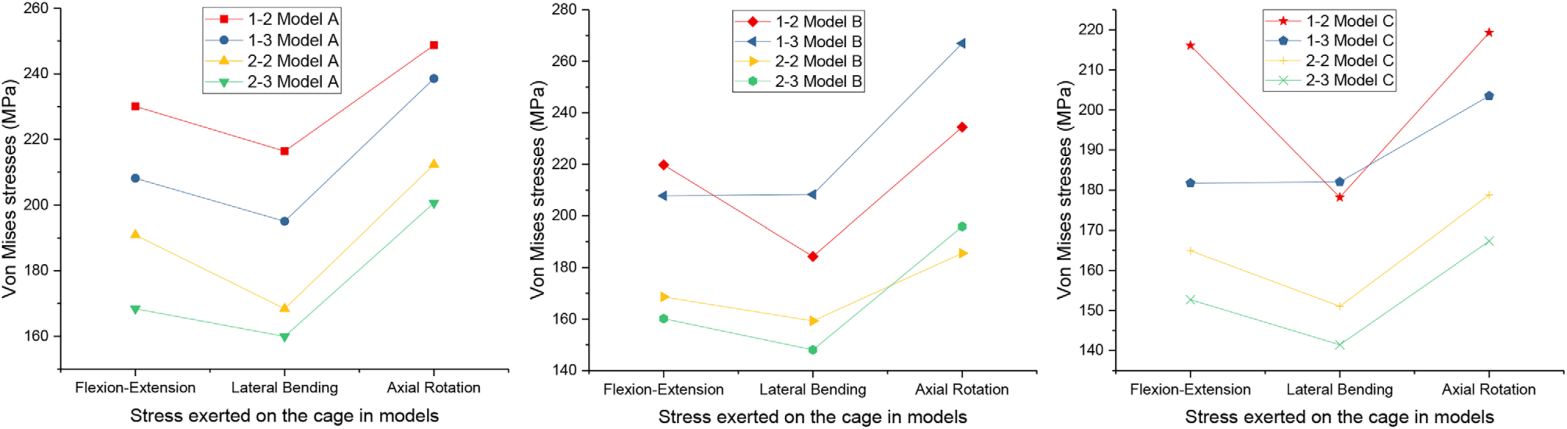
Maximum von Mises stress (MPa) in the interbody cage for implanted models

The maximum von Mises stress in the interbody cage for the implanted models is shown in Figure 9. For Models A, B, and C, the stress of the cage in types 2–2 and 2–3 was lower than that of types 1–2 and 1–3 under all loading conditions. Although the cage stress in type 2–3 (195.91 MPa) was slightly higher than that of type 2–2 (185.57 MPa) in terms of the axial rotation in Model B, the difference was not significant. Overall, the cage stress in type 2–3 was less than that in type 2–2. For Models A, B, and C, the minimum stress of types 2–2 and 2–3 was under lateral bending, and the maximum stress was under axial rotation.

### Magnitudes of the Maximum von Mises Stress at the Screw‐Bone Interface

**Fig. 10:**
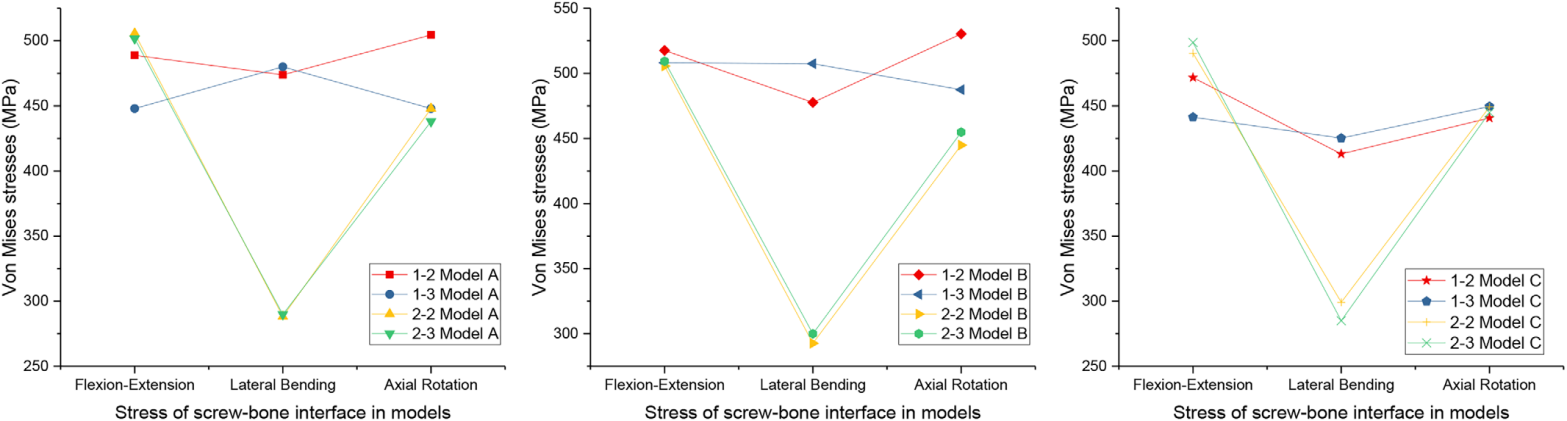
Maximum von Mises stress (MPa) of screw‐bone interface for implanted models

The peak stress at the screw‐bone interface was also investigated. It could reflect the load distribution between the vertebrae and the spinal implants. It is important to assess the risk of screw loosening and migration at the fusion segment, one of the important complications of internal fixation. Figure 10 displays the maximum von Mises stress of the screw‐bone interface for the implanted models. In terms of flexion‐extension and axial rotation, the models did not differ significantly under any loading condition. However, the peak stresses at the screw‐bone interface in types 2–2 and 2–3 were much lower than those in types 1–2 and 1–3 under lateral bending. In Models A and B, the difference even reached 200 MPa, while in Model C, the difference was also approximately 150 MPa. Meanwhile, under all loading conditions, the peak stresses of types 2–2 and 2–3 at the screw‐bone interface were basically the same in all of the models. The minimum stress of types 2–2 and 2–3 was observed under lateral bending, the maximum stress was observed under flexion‐extension, and the difference was approximately 200 MPa. The stress nephograms of the *in situ* screws of four types in different models under all loading conditions are shown in the supplemental materials.

### Magnitudes of the Maximum von Mises Stress of the L4 Superior Endplate

**Fig. 11:**
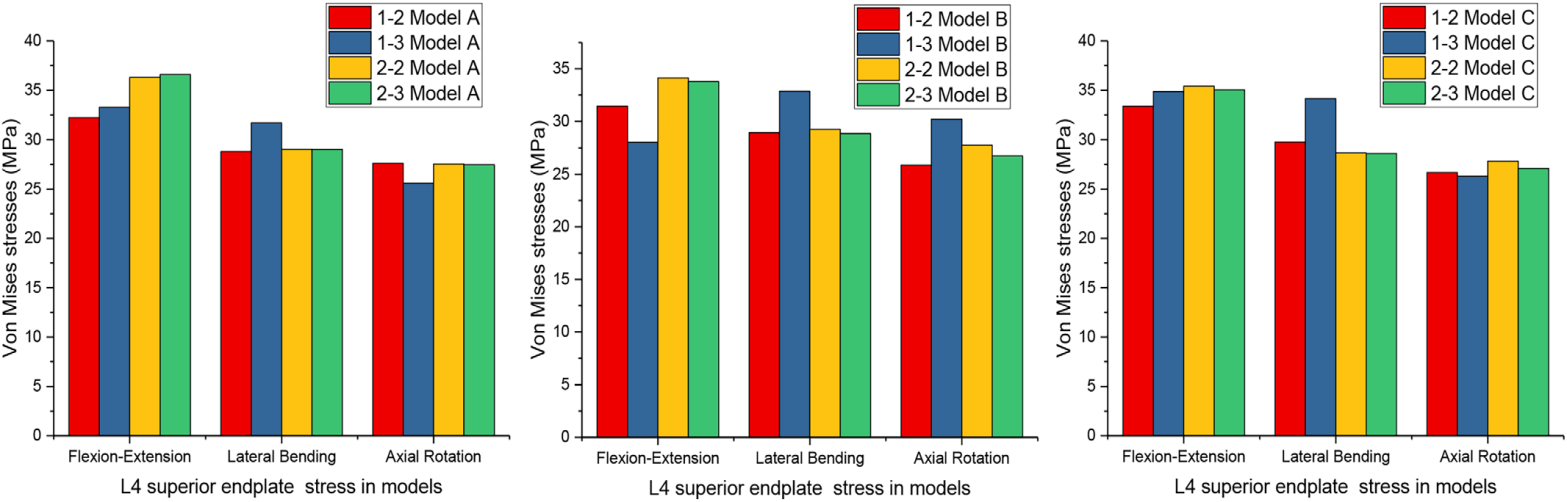
Maximum von Mises stress (MPa) of the L4 superior endplate for implanted models

The peak stress of the L4 superior endplate, which was associated with endplate fracture and cage subsidence, was also investigated, as shown in Figure 11. Apart from types 1–3 in Model B, the stress of the L4 superior endplate in all models was largest under flexion‐extension and lowest under axial rotation, but the differences were not significant. The stresses of types 2–2 and 2–3 were slightly larger than those of types 1–2 and 2–2 in terms of flexion‐extension in all models. The peak stress of the L4 superior endplate was approximately 30 MPa in all models under all loading conditions, and the difference between the largest and the lowest was within 10 MPa in the same model and loading condition.

## Discussion

### *Stability of Anterolateral* In Situ *Screw Fixation Following LLIF*

Our study showed that the ROM of all models with implants was restricted under all loading conditions compared with the intact model, and the lowest ROM restriction was under flexion‐extension, while the largest was under lateral bending. In terms of the screw implantation angle in the coronal plane, the difference in ROM restriction was not significant among the different models. From the aspect of the screw implantation region in the horizontal plane, types 2–2 and 2–3 were restricted more than types 1–2 and 1–3, with no significant difference. The maximum stress of the interbody cage in types 2–2 and 2–3 was approximately 20% lower than that in types 1–2 and 1–3 under all loading conditions. For the peak stress of the screw‐bone interface, the models did not differ significantly in terms of flexion‐extension and axial rotation. However, in types 2–2 and 2–3, it was much lower (40%, 40%, 30% in Models A, B, C, respectively) than that in types 1–2 and 1–3 under lateral bending.

Previous studies have reported that stand‐alone LLIF was insufficient to provide stability under any loading condition, and this resulted in various complications^27^, ^28^, ^29^. Therefore, supplementary internal fixation is now performed in LLIF surgery. Posterior internal fixation with pedicle screws is thought to have an excellent capacity to stabilize the fused segment due to the high stiffness of the structure and thus has been widely used in the field of spinal surgery. Compared to the posterior approach, *in situ* screw fixation through the LLIF incision can avoid changes in the patient's position during the operation, the need for a second incision, and injury to the spinal cord and nerve by the pedicle screws^30^. Furthermore, previous biomechanical studies have demonstrated that lateral instrumentation has a good ability to enhance the overall stability and has reasonable biomechanical protection, similar to pedicle screws^8^. To further investigate the influence of different regions and angles of *in situ* screw implantation on the stability of the fused segment, our study first used FEA to explore screw‐setting strategies for anterolateral *in situ* screws following LLIF from a biomechanical point of view.

### *Biomechanical Effect on* In Situ *Screw Fixation Following LLIF*

Less ROM does not necessarily mean more stability. A stable system is one that does not undergo large displacements under small perturbations. According to the FDA definition, a less than 5° ROM is considered to be a successful fusion in clinical practice^31^. In our study, the results showed that the maximum ROM of all models was only 4.2°, which again confirmed that supplemented *in situ* screw fixation could effectively offer sufficient stability for the segment fused in LLIF surgery. In addition, the *in situ* screw effectively reduced the ROM under lateral bending and axial rotation, and the effect was the most obvious in the former condition. These results are consistent with the findings of Shasti *et al*., who found that the reduction in lateral bending ROM was more remarkable when supplemented by an *in situ* screw in LLIF^32^. Since biomechanical studies cannot simulate the fusion process, the ROM was chosen for the comparisons. Xu *et al*. also believed that ROM was an external response that was sensitive to the material properties of the spine^14^.

Although all reconstructive models increased the stability of the fused segment compared to the intact model under all loading conditions, their magnitudes were not the same. Our results showed that types 2–2 and 2–3 had less ROM restriction than types 1–2 and 1–3 in flexion‐extension. In contrast, under both lateral bending and axial rotation, types 2–2 and 2–3 had more ROM restriction than types 1–2 and 1–3. Types 2–2 and 2–3 were more similar than types 1–2 and 1–3. Moreover, in the same implantation region, there was no significant difference in the stability of Models A, B, and C. Therefore, we believe that the stability of the fused segment is not correlated with the implantation angle of the *in situ* screw. Instead, the difference might be caused by different regions of implantation in the horizontal planes. These findings are only useful for describing the static effect on immediate postoperative stability, but they do reflect an overall trend.

In clinical practice, screw loosening and breakage are common reasons for revision of internal spinal fixation. Reducing the stress on the screws could reduce the risk of screw breakage and loosening^33^, ^34^, ^35^. Therefore, we also investigated the peak stress in the interbody cage and screw‐bone interface in Models A, B, and C with different implantation regions. In our study, there was no significant difference in the stress placed on the screw‐bone interface of various types in terms of flexion‐extension and axial rotation. Under lateral bending, the stresses of types 2–2 and 2–3 were significantly less than those of types 1–2 and 1–3. Under all loading conditions, the highest stress of types 2–2 and 2–3 could be found at the screw‐bone interface, where the peak stress reached approximately 500 MPa under flexion‐extension, 300 MPa under lateral bending, and 300 MPa under axial rotation. The *in situ* screw is made of titanium alloy, and its typical mechanical properties are 1380–2070 MPa for ultimate bearing strength and 825–895 MPa for yield strength^36^. Judging from the above data, the stress on the *in situ* screw was in the range between the yield strength and ultimate strength.

Cage subsidence with endplate injury is another complication that can occur after spinal fusion surgery. Although severe osteoporosis is a clear risk factor, the effect of stress on the cage should also be considered. From the aspects of biomechanics, excessive stress on the interbody cage could lead to cage subsidence. In our study, for Models A, B, and C, the stress on the cage in types 2–2 and 2–3 was lower than that in types 1–2 and 1–3 under all loading conditions. In addition, the stress in type 2–3 was less than that in type 2–2. Therefore, types 2–2 and 2–3 might have relatively low incidences of cage subsidence and endplate injury. Of note, although type 2–3 showed the advantages of less stress on the screw‐bone interface compared with type 2–2 from the perspective of biomechanics, it required more technical skill by the spinal surgeons for implantation and might increase the risk of encroachment on the foramina and spinal canal in clinical practice.

In addition, endplate stress is also associated with endplate injury and cage subsidence. Previous biomechanical and clinical studies have demonstrated that supplemental pedicle or lateral screw fixation could significantly decrease endplate stress and effectively reduce the incidence of cage subsidence with endplate injury^37^, ^38^, ^39^, ^40^. However, the effect of the implantation region and angle on endplate stress remains unclear. Therefore, we investigated the maximum stress of the L4 superior endplate. Our study showed that the stress was largest under flexion‐extension and lowest under axial rotation in all models (apart from types 1–3 in Model B, the largest was under lateral bending and the lowest was under flexion‐extension). In terms of flexion‐extension, types 2–2 and 2–3 showed slightly more stress than types 1–2 and 2–2 in all models. Although differences between different types and conditions did exist, they were not statistically significant. Hence, it appeared that different lateral screw implantation regions and angles had little effect on protecting the endplate. This result may be due to lateral screw implantation increasing the stiffness of the global VB rather than the stiffness of a limited area on the endplate.

### Validation and Stress Sensitivity Analysis of the FE Model

The intact finite element model was validated in this study, which was in good agreement with the results of previous studies. Stress sensitivity analysis can provide accurate insight into FE models investigating stress or strain. Our results showed that the 25% difference in modulus led to a total difference in ROM of 13.8% and 21.5% and IDP of 9.9% and 13.4% under flexion and compression, respectively. Actually, the tendency of the predicted results under various fixation options would not be materially changed depending on the individual geometric model and simplified material properties. Therefore, the intact FE model in this study was reasonable and sensitive for investigating biomechanical behavior. Of course, since there are many parameters of the material properties of the lumbar spine, a stress sensitivity analysis could not compare the individual effects of each parameter on the biomechanical behavior. In future research, we will analyze each parameter of the spinal components in detail.

### Limitations

Although the findings in this study might be meaningful for clinical practice, its limitations should not be ignored. Bone tissues, ligaments, and implants all have linear‐elastic material properties. Since the focus of this study is not to predict the mechanical behavior of implants, isotropic linear‐elastic material models can be used to simulate the preyield mechanical behavior^41^, ^42^. Many FEA studies of the lumbar spine have assumed that the components of the spine are linear to simplify the calculations^43^, ^44^, ^45^. Although this study was not a clinical trial, it is much better for informing professional practice than simply using information, opinions, or data without an implied degree of accuracy. Additional clinical studies will be conducted to evaluate the results of this study in the future.

## Conclusion

This study found that the angle of the *in situ* screws had little effect on the stability of LLIF. Types 2–2 and 2–3 could both provide better mechanical stability under all loading conditions and provide superior protective effects on the interbody cage and screw‐bone interface under lateral‐bending conditions. Compared with type 2–2, type 2–3 might have a higher risk of injury to the spinal cord or nerve. Therefore, the lateral middle region of the VB can be selected as the entry point for *in situ* screws in clinical practice. Moreover, type 2–2 might be a safer implantation strategy for *in situ* screws. Of course, additional clinical studies are needed to evaluate and confirm these findings.

## CONFLICT OF INTEREST

No benefits in any form have been received or will be received from a commercial party related directly or indirectly to the subject of this article.

## Supporting information


**Table S1.** The material properties of spinal components
