# Supplementary material for: Effect of the In Situ Screw Implantation Region and Angle on the Stability of Lateral Lumbar Interbody Fusion: A Finite Element Study
Source: Orthop Surg. 2022 Jun 3;14(7):1506–17. doi: 10.1111/os.13312 (PMC9251290; doi:10.1111/os.13312)
Supplement: Supplementary file 1 — Table S1. The material properties of spinal components [file OS-14-1506-s001.docx]

**Supplementary materials**

***Part one***


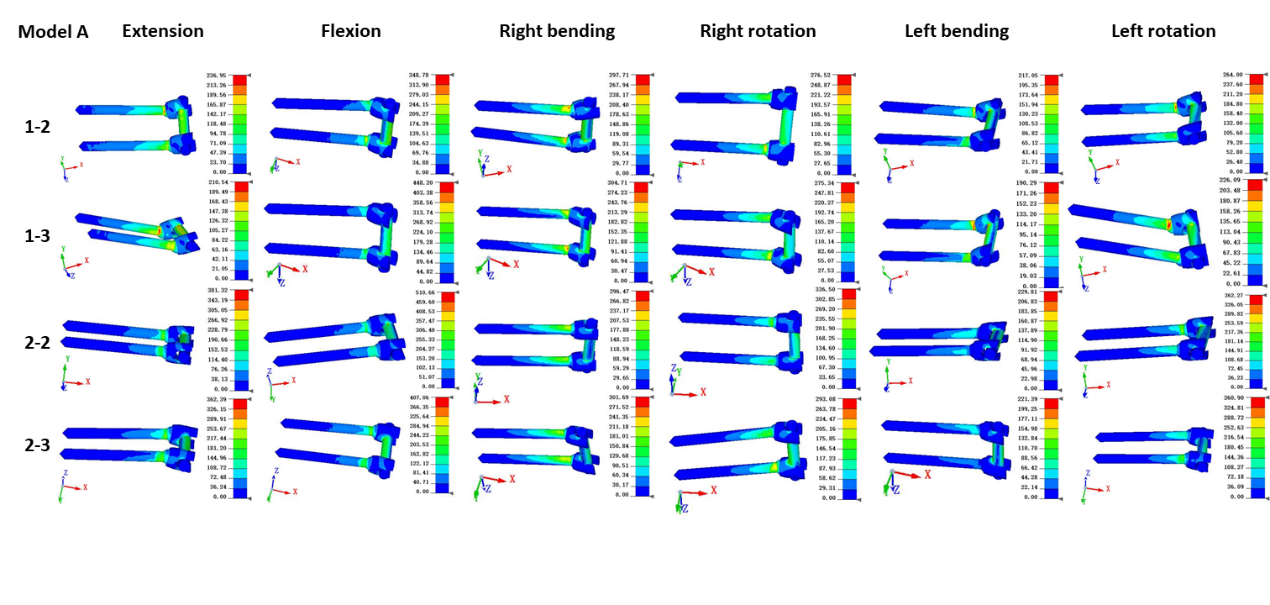


The stress nephogram of in situ screw of four types in Model A under all loading conditions.


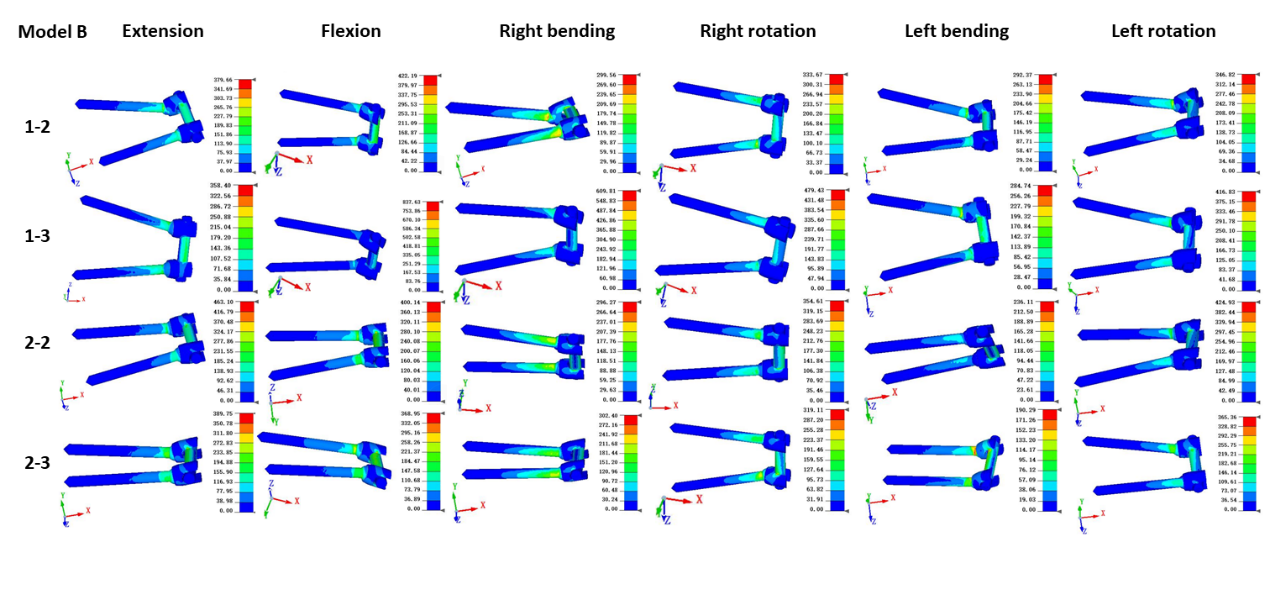


The stress nephogram of in situ screw of four types in model B under all loading conditions.


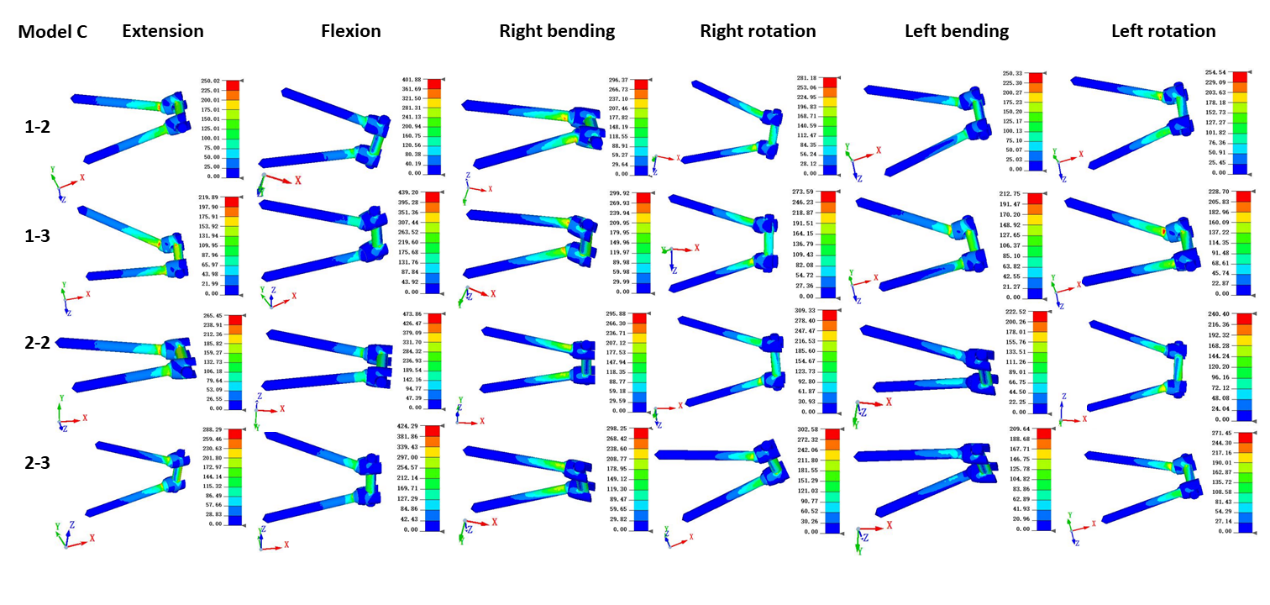


The stress nephogram of in situ screw of four types in model C under all loading conditions.

**Note**: In the coordinate system at the left bottom, the Z axis always points to the upper screw, the Y axis points to the back and the X axis points to the right side.

***Part two***

Table 1 The material properties of spinal components

| Element set | Young modulus（MPa） | Poisson ratio | Element type | Thickness (mm) |
| --- | --- | --- | --- | --- |
| Cortical bone | 12000 | 0.3 | 4-node shell element | 1 |
| Cancellous bone | 100 | 0.2 | 8-node solid element | / |
| Posterior bony elements | 3500 | 0.25 | 8-node solid element | / |
| Cartilaginous endplate | 23.8 | 0.4 | 4-node shell element | 0.8 |
| Nuclear pulposus | 1 | 0.495 | 8-node solid element | / |
| Annulus fibers: | 495 | 0.3 | Truss element | / |
| Annulus substance  Spinal Ligaments: | 4.2 | 0.45 | 8-node solid element | / |
| ALL | 7.8 | 0.3 | 4-node shell element | 2.4 |
| PLL | 10 | 0.3 | 4-node shell element | 1.1 |
| LF | 15 | 0.3 | 4-node shell element | 3.0 |
| SSL | 8 | 0.3 | 4-node shell element | 1.7 |
| ISL | 10 | 0.3 | 4-node shell element | 1.7 |
| ITL | 10 | 0.3 | 4-node shell element | 1.2 |
